# Threat of attacks of *Ixodes ricinus* ticks (Ixodida: Ixodidae) and Lyme borreliosis within urban heat islands in south-western Poland

**DOI:** 10.1186/s13071-014-0562-y

**Published:** 2014-12-11

**Authors:** Alicja Buczek, Dariusz Ciura, Katarzyna Bartosik, Zbigniew Zając, Joanna Kulisz

**Affiliations:** Chair and Department of Biology and Parasitology, Medical University, Radziwiłłowska 11, 20-080 Lublin, Poland

**Keywords:** *Ixodes ricinus*, Tick abundance, Tick activity, Urban heat islands, Environmental pollution, Borreliosis

## Abstract

**Background:**

The increased incidence of Lyme disease in Europe necessitates permanent monitoring of the occurrence and activity of its vector. Therefore, in this study, we have investigated the presence and seasonal activity of *Ixodes ricinus* ticks in various habitats of a large industrial region in south-western Poland in correlation with environmental factors present in urban heat islands. Additionally, the risk of borreliosis in this part of Poland has been assessed.

**Methods:**

The investigations were carried out at two-week intervals between April and October 2001 and 2002. Ticks were collected from four localities in Upper Silesia, i.e. in a city park (CH), on the outskirts of two large cities (KO, MI), and in a forest (KL). We analysed the impact of temperature and humidity measured during the collection period in the *I. ricinus* habitats, the climatic conditions prevailing in the study area, and the degree of environmental contamination on the abundance and activity of these ticks in the respective sites. The degree of borreliosis risk in the region was determined on the basis of the results of research on the prevalence of *Borrelia burgdorferi* s.l. in ticks and reports from sanitary-epidemiological stations.

**Results:**

In total, 2061 *I. ricinus* ticks, including 606 nymphs and 1455 adults, were collected in the study area. The number and activity of the ticks varied during the collection in the different sites. In the urban locality CH, tick abundance was the lowest (455 throughout the investigation period), and the seasonal activity of females was unimodal and persisted for as long as 4 months. In the suburban localities KO and MI, tick abundance was higher (485 and 481 specimens, respectively) and the activity of females was unimodal. The highest abundance (640 ticks) and a bimodal pattern of female activity were reported from the forest locality KL. In all the localities, the activity of nymphs was unimodal. Humidity was found to be a factor influencing *I. ricinus* abundance and activity, whereas temperature did not affect their number and behaviour significantly. The climate parameters within the urban heat islands noted during the investigations contributed to dispersal of dust and gas pollutants. The analysis of the data reveals that there is a risk of borreliosis in the entire study area; however, it is higher in the urban localities than in the suburban sites.

**Conclusions:**

Environmental conditions (habitat, climate, and dust and gas pollution) prevailing within urban heat islands may exert an impact on tick abundance and activity and the prevalence of Lyme disease in the study area. The greatest effect of the environmental factors on ticks was found in the city park, where the risk of human infection with *B. burgdorferi* s.l. spirochetes is the highest as well.

## Background

The wide distribution of *Ixodes ricinus* ticks in Europe and the diversity of their habitats pose a threat of tick attacks to humans in urban and suburban areas. Mechanical skin damage caused by tick mouth organs and biologically active substances introduced with saliva during feeding trigger local and systemic reactions in hosts [1,2], including life-threatening anaphylactic shock in humans. Even greater importance is ascribed to the ability of ticks to transmit many pathogens, e.g. the spirochete *Borrelia burgdorferi* sensu lato complex, i.e. the aetiological agent of Lyme borreliosis with the greatest relevance for public health. Among the 19 species belonging to this complex, three genospecies *B. garinii, B. afzelii*, and *B. burgdorferi* s.s., which differ in tissue tropism and host preferences, are regarded as the major pathogenic factors in Europe.

Borreliosis is a multisystem disease occurring in three clinical forms, i.e. early localised, early disseminated, and late infection [3]. It typically manifests in a variety of dermatological, osteoarticular, neurological, and cardiac symptoms [4-6]. The development of borreliosis in humans is determined by the general condition of the immune system and the degree of invasiveness of the spirochete strain.

Despite the intensive multi-faceted ongoing research into this zoonosis, in clinical practice the disease still poses many diagnostic and interpretation problems that are becoming more prevalent worldwide. The sources of the problems include, on the one hand, the expanding distribution range of borreliosis vectors, i.e. ticks from the *I. ricinus-persulcatus* complex and, on the other hand, the increased interest in tourism and recreation resulting in expanding human presence in tick habitats, and the rising numbers of immunosuppressed people. These factors substantiate the need for monitoring the occurrence and activity of Lyme disease vectors in different environments and the prevalence of tick-borne pathogens in both ticks and humans.

This study presents the abundance and activity of *I. ricinus* ticks and assessment of the threat posed by *B. burgdorferi* s.l. spirochetes in various habitats of the most industrialized region in Poland in relation to the conditions prevailing within urban heat islands.

## Methods

### Study sites

The investigations were carried out in four sites located in the area of Upper Silesia (19°00'E, 50°15'N).

1. The Provincial Park of Culture and Recreation in Chorzów (CH) with an area of ca. 506 ha is situated within a post-industrial wasteland. It is surrounded by high urban developments of three cities: Katowice, Chorzów, and Siemianowice. The site has varied terrain relief and diverse habitat conditions (woodland, grassland, wetland, and aquatic areas). The area of tick collection is dominated by oak, poplar, maple, beech, birch, hazel, linden, spruce, and fir trees; the shrub layer comprises bilberry, bird cherry, viburnum, thuja, and buckthorn, and the undergrowth is dominated by ferns and grasses.
2. Ochojec (KO) - a locality in Katowice in a slightly undulating area of the south-eastern part of the city near residential districts and a forest community dominated largely by alder, oak, beech, and pine trees as well as forest-scrub (alder-willow) and meadow communities. The brushwood is overgrown by buckthorn and red oak, and the undergrowth comprises grasses, ferns, and bilberries.
3. The locality near Mikołów (MI) is situated on the north-eastern city outskirts within a forest complex composed of forest, forest-scrub, marshland, and grassland communities. The mixed forests covering the study area are dominated by birch, alder, aspen, and oak trees. The admixture contains pine and spruce, the brushwood is dominated by buckthorn and rowan, and the undergrowth by grasses, ferns, and bilberry.
4. Ligota (KL) - a locality in Katowice situated on a plain area near the southern district of the city. It is dominated by natural and semi-natural plant communities, primarily forest-scrub and meadow associations. The forests are dominated by oak, alder, beech, and pine trees, and the admixture contains birch and spruce. The brushwood comprises abundantly growing buckthorn and the undergrowth is dominated by bilberry and grass.

The sites are located in areas that are frequently visited by the residents of Upper Silesia and tourists from Poland as well as forestry workers and employees of the recreation centres.

### Tick collection

*I. ricinus* (nymphs and adult stages) were collected with the flagging method between April and October 2001 and 2002 during the peak diurnal activity period for this species, i.e. between 15:00 and 18:00. A 1 m^2^ flannel cloth attached to a 1.5-m bamboo pole was used for sweeping plants and plant litter. After a few movements of the cloth, tick specimens attached thereto were collected and transferred into tubes filled with 70% alcohol. In each site, ticks were collected at 2-week intervals, each time for a period of 1 hour by one person. There was no tick collection on rainy days or after heavy rainfall. Simultaneously, during each collection round, electronic devices were used to measure humidity with an accuracy of 1% and temperature with an accuracy of 1°C.

During the investigations of tick activity, particular attention was focused on nymphs and females, as these infest humans most frequently.

### Environmental data

Data on environmental pollution reported from the Upper Silesia agglomeration during the investigations were obtained from the Regional Inspectorate for Environmental Protection in Katowice [7]. Information about the climatic conditions prevailing in the respective months of 2001 and 2002 provided by meteorological station reports was included in the study [7].

### Epidemiological analyses

Data on the prevalence of *B. burgdorferi* s.l. and genospecies in ticks [8] were used for assessment of borreliosis risk in the study areas. Data on the incidence of Lyme disease among the residents of Upper Silesia and Poland in the period of 2000-2012 was obtained from National Institute of Hygiene reports [9].

### Statistical analysis

The statistical analysis of the results was based on the two dependent sample means t-test to determine the differences between tick abundance in 2001 and 2002. The analysis of variance F-test was employed for comparison of the tick abundance in the different collection rounds (half of the months), and the Friedman test for multiple dependent samples was used to compare the abundance of the individual tick stages and tick abundance in the four localities.

The analysis of the correlations between tick abundance and temperature and humidity was based on Pearson's correlation coefficient, which indicates the strength and direction of the correlations between the traits studied. A 5% inference error was accepted. The level of p < 0.05 indicates statistically significant differences or correlations. The calculations and graphs were made using STATISTICA and Microsoft Excel programs.

## Results

In total, 2061 *I. ricinus* (606 nymphs and 1455 adult stages) were collected in the study area. The number of ticks and their activity differed between the four sites during the two years of the investigations. The greatest number of questing ticks (640 specimens, including 42% of nymphs and 58% of adults) were collected in the forest locality in Katowice Ligota. Lower numbers were found in the suburban localities in Katowice Mikołów (481 specimens, including 32% of nymphs and 68% of adults) and Ochojec (485 specimens, including 24% of nymphs and 76% of adults), and in the urban park in Chorzów (455 specimens, including 15% of nymphs and 85% of adult stages).

**Figure 1:**
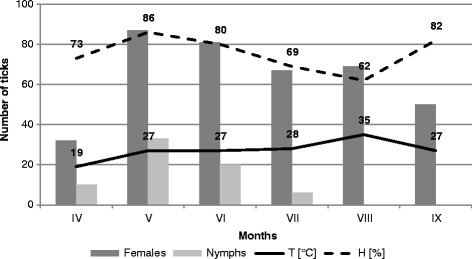
**Activity of nymphs and females of** ***Ixodes ricinus*** **in the urban locality in Chorzów (CH).**

**Figure 2:**
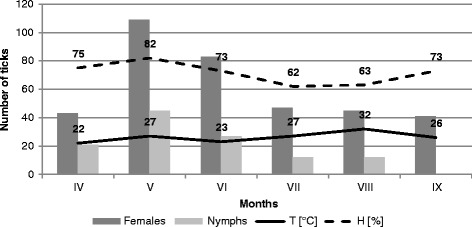
**Activity of nymphs and females of** ***Ixodes ricinus*** **on the outskirts of the city in Ochojec (KO).**

**Figure 3:**
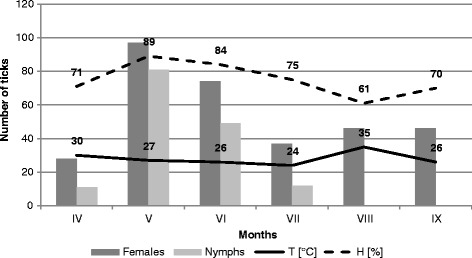
**Activity of nymphs and females of** ***Ixodes ricinus*** **on the outskirts of the city in Mikołów (MI).**

**Figure 4:**
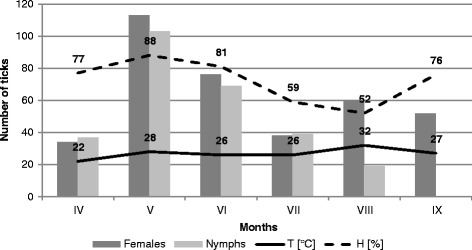
**Activity of nymphs and females of** ***Ixodes ricinus*** **in the forest habitat in Ligota (KL).**

In the site located in the forest ecosystem, the collected nymphs accounted for a significantly higher proportion (36.82% and 42.61% in 2001 and 2002, respectively, in Katowice Ligota) than in the other localities. Throughout the study period, 64.75 ± 4.74 nymphs on average were collected in this area within one hour (Table 1). The lowest number of nymphs collected per hour (17.25 ± 2.09) was reported from the city park in Chorzów. The proportion of nymphs collected in this locality in 2001 and 2002 was 5.03 and 23.6%, respectively. The statistical tests confirmed the differences in the nymph abundance in the studied sites (chi^2^ = 16.139, p = 0.001). Nymph activity in the forest habitat of Ligota (F = 2.841, p = 0.060), and in the suburban sites in Ochojec (F = 0.725, p = 0.680) and Mikołów (F = 0.869, p = 0.579) persisted at a similar level in the collection months during both study years. The activity of nymphs in all the localities was unimodal (Figures 1, 2, 3 and 4).

**Table 1:** **Frequency of occurrence of various** ***Ixodes ricinus*** **stages in the different localities during 1 h collection**

**Developmental stage**	**Investigation year**	**CH**	**KO**	**M**	**KL**	**Total study area**	**Statistical comparison of results**
**Nymphs**	2001	5.01 ± 1.05	10 ± 2.11	5.0 ± 1.05	51 ± 4.03	71.0 ± 6.54	X^2^ = 16.811 ; p = 0.001
	2002	29.5 ± 3.36	48.5 ± 3.42	71.5 ± 8.22	75 ± 6.51	153 ± 11.99	X^2^ = 5.923 ; p = 0.115
	Total	17.25 ± 2.09	29.25 ± 2.19	38.25 ± 4.52	64.75 ± 4.74	149 ± 50.0	X^2^ = 16.139 ; p = 0.001
**Males**	2001	51.0 ± 1.51	50.0 ± 1.80	33.50 ± 1.72	33.5 ± 1.83	168 ± 5.73	X^2^ = 17.625 ; p = 0.001
	2002	47.5 ± 1.57	48.5 ± 2.31	46.0 ± 2.04	47.5 ± 2.32	189.5 ± 6.09	X^2^ = 1.181 ; p = 0.758
	Total	49.25 ± 0.97	49.25 ± 1.87	39.75 ± 1.73	38.5 ± 1.99	176.75 ± 5.65	X^2^ = 8.394 ; p = 0.039
**Females**	2001	43.5 ± 1.75	47.0 ± 2.63	36.0 ± 1.93	54.0 ± 2.82	180.5 ± 7.9	X^2^ = 6.719 ; p = 0.081
	2002	48.0 ± 1.34	38.5 ± 2.32	50.5 ± 3.05	53.5 ± 3.21	190.5 ± 8.98	X^2^ = 4.906 ; p = 0.179
	Total	45.75 ± 1.07	42.75 ± 2.24	43.25 ± 2.33	54.75 ± 2.61	186.5 ± 7.9	X^2^ = 10.832 ; p = 0.01

CH the urban locality in Chorzów, KO - the outskirts of the city in Ochojec, MI- the outskirts of the city in Mikołów , KL- the forest habitat in Ligota.

Adult stages dominated in all the tick collection rounds, but the proportion of females and males differed between the studied sites in 2001 and 2002 (ch^2^ = 10.832, p = 0.013 and ch^2^ = 8.394, p = 0.039, respectively) (Table 1). The activity of female and male *I. ricinus* ticks assessed on the basis of the number of specimens collected per hour were statistically significantly different between the localities in Ligota (F = 4.532, p = 0.014 and F = 3.162, p = 0.044, respectively), Ochojec (F = 4.205, p = 0.018 and F = 5.066, p = 0.09, respectively), and Mikołów (F = 3.597, p = 0.029 and F = 3.411, p = 0.035, respectively) during the different months (Figures 1, 2, 3 and 4). In turn, no statistically significant differences in the activity of females and males during the seasonal activity of this species were reported from the Chorzów site (F = 0.9666, p = 0.516 and F = 0.724, p = 0.681).

In the forest KL locality, a bimodal *I. ricinus* female activity was noted, which was higher in May and twice as low in the second half of August and early September. In turn, in the urban site (CH) and the two suburban sites (KO and MI), females exhibited a unimodal pattern of activity. However, in the city park, the unimodal tick activity persisted throughout the study period between the first half of May and the first half of September (Figures 1, 2, 3 and 4).

A comparison of the investigation results obtained in the different localities shows statistically significant differences in the number of nymphs and males (p = 0.001) collected in the individual months of 2001 and throughout the study period and females collected throughout the study period (p = 0.001 and p = 0.013, respectively) (Table 1). Significant differences in the numbers of collected specimens, irrespective of the developmental stage, between the localities were found in 2001 (p = 0.004) and throughout the study period (p = 0.007).

Of the two parameters measured during the field study, humidity had a statistically significant effect on the abundance and activity of nymphs, females, and males in the suburban localities. In the urban park, humidity had a significant impact only on the abundance and activity of females and all ticks (Figures 1, 2, 3 and 4). In all the sites and area analysed, no relationship was found between the temperature prevailing in tick habitats and tick abundance and activity.

According to the meteorological data for the period between January and the end of September, the mean values of temperature and wind speed in our study area were higher in the individual months of 2002, while the humidity values were lower than those in 2001 (Table 2).

**Table 2:** **Meteorological conditions in the study area in 2001 and 2002**

	**Katowice 2001**											
**Weather parameters**	**Months**											
	**I**	**II**	**III**	**IV**	**V**	**VI**	**VII**	**VIII**	**IX**	**X**	**XI**	**XII**
Average temp. (°C)	-0.5	0.6	3.6	7.9	14.6	19.3	14.6	19	11.8	11.7	2	-5.1
Max temp. (°C)	2.4	4.7	7.7	12.8	20.5	24.2	19.3	24.9	15.6	16.5	5.1	-1.3
Min temp. (°C)	-4.1	-4.2	-0.9	2.6	7.5	14.0	9.4	13.1	8.0	7.0	-1.6	-9.7
Average air pressure (hPa)	1019	1017	1010	1013	1016	1014	1014	1017	1012	1020	1019	1021
Average RH (%)	87.0	77.0	79.1	73.4	63.8	77.4	77.1	75.3	86	84.5	85.1	89.2
Average wind speed(m/s)	8.2	10.5	10.7	8.6	8.4	7.3	8.0	7.1	10.0	7.8	10.8	11.2
Average rainfall (mm)	-	31.2	60.1	102.2	65.3	98.2	151.5	101.7	109.9	31.4	55.5	37.5
	**Katowice 2002**											
**Weather parameters**	**Months**											
	**I**	**II**	**III**	**IV**	**V**	**VI**	**VII**	**VIII**	**IX**	**X**	**XI**	**XII**
Average temp. (°C)	-0.8	4.2	5.3	8.8	17.0	16.4	19.6	19.4	12.3	7.2	5.2	-5.1
Max temp. (°C)	2.2	8.4	10.7	14.1	23.4	22.1	26.1	25.6	17.6	11.5	9.5	-1.3
Min temp. (°C)	-4.8	-1.0	-0.3	2.5	9.4	10.6	13.3	13.3	7.1	2.9	0.8	-9.7
Average air pressure (hPa)	1025	1014	1018	1017	1015	1015	1016	1014	1017	1013	1012	1021
Average RH (%)	82.4	76.2	69.5	65.5	64.0	75.7	70.4	76.6	79.6	84.7	85.4	89.2
Average wind speed (m/s)	12.7	13.9	12.1	9.5	7.8	9.3	10.5	8.1	9	14.1	11.3	11.2
Average rainfall (mm)	39.7	47.8	21.8	22.5	133.8	132.2	91.3	82.6	55.9	83.1	36.9	25.3

The data presented in Table 3 show that dust and gas emissions in the Upper Silesia agglomeration in 2001-2002 persisted at a high level and, according to the health care criteria, exceeded the acceptable standards for the investigated area [9].

**Table 3:** **Air pollution in the study area in years 2001-2002** [7]

	**2001**	**2002**
**Dust emissions**	**32,8 thousand mg**	**30,5 thousand mg**
**Gas emissions, including**:	**33997,3 thousand mg**	**37737 thousand mg**
Sulphur dioxide	144,95 thousand mg	149,3 thousand mg
Nitrogen oxides	76,3 thousand mg	75,6 thousand mg
Carbon oxide	125,1 thousand mg	142,8 thousand mg
Carbon dioxide	33523,7 thousand mg	37134,3 thousand mg
Other: methane, hydrocarbons	no data	234,8 thousand mg

**Figure 5:**
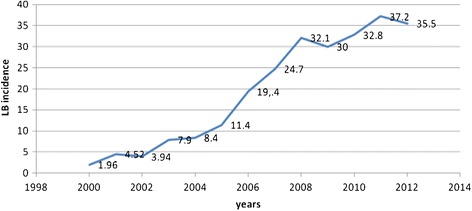
**Incidence of Lyme disease (LB) in Upper Silesia in years 2000-2012 [** 9 **]** **.**

Over the last 12 years, the incidence of Lyme disease in Upper Silesia has increased (Figure 5). In 2007-2012, it ranged between 24.7 and 38.0 per 100 000 inhabitants, which places the region at the 10 position in Poland in terms of the incidence of this zoonosis [10]. The greatest numbers of borreliosis cases were recorded in the third and fourth quarter of each year.

## Discussion

The abundance and activity of *I. ricinus* have been investigated many times in various regions of the geographic distribution of the species. The investigations were primarily focused on the effect of temperature and humidity, vegetation, and topography of the terrain [11-17] on the abundance and dynamics of the activity of this tick species. Yet, no research has been focused on the conditions of urban heat islands.

The urban heat island is a dynamic phenomenon characterised by diurnal and annual variability. The greatest differences in temperature and humidity between urban and non-urban areas are observed during sunny and cloudless days. The form and intensity of the urban heat island are a result of interactions between various processes, e.g. the radiation balance of the city related to air pollution and the geometry of urban structures, the heat capacity of building materials depending on the thermal properties and humidity of soil surrounding the city, as well as materials used for construction of artificial surfaces in the city [18-20]. The climate of the city is influenced by a variety of factors that depend on the structure of the city, the physical characteristics of the area, and meteorological conditions [20].

The Silesian Province is one of the Polish regions with the highest levels of dust (ca. 22% of national emissions) and non-carbon dioxide gas emissions (ca. 24%) (Table 3), which may affect the abundance and questing behaviour of ticks inhabiting the area.

Likewise in the urban park (CH) and in the suburban localities of the large agglomeration (KO and MI) where *I. ricinus* abundance was reported to be lower than in the forest habitat (KL), a decline in the abundance of these ticks was also observed in other industrial regions. Siuda et al. [21] explain the decreased abundance of *I. ricinus* populations in southern Poland by the impact of chemical contamination transported with air currents from large industrial districts, e.g. Upper Silesia and Cracow (south-western and southern Poland). A comparative study carried out by Černy *et al.* [22] near the city of Most in an industrial region in the north-western Czech Republic and in less degraded areas of the central Czech Republic demonstrated a large impact of anthropopressure on *I. ricinus* abundance. A teratogenic effect of chemicals on tick development has been evidenced by an increased prevalence of morphological abnormalities in specimens collected in heavily polluted environments [23-27].

The level of pollution is largely dependent on meteorological conditions, e.g. atmospheric pressure, temperature, precipitation, and speed and direction of wind, which are associated with the type of urban development [20]. High-pressure weather, which was recorded during our field observations, promotes an increase in the concentration of pollutants (Tables 2 and 3).

As shown in our investigations, *I. ricinus* abundance and activity were strongly influenced by humidity of the tick habitats, which ranged from 56% to 94% during the collection periods. According to the data recorded by meteorological stations, the average air humidity in the spring months throughout the study period ranged from 63.8% to 87% and, in comparison with 2001, was lower in 2002 at a higher average air temperature. These differences in the humidity and temperature conditions may have changed the rhythm of *I. ricinus* seasonal activity in both years of our study.

A unimodal pattern of the seasonal activity of *I. ricinus* females was observed in the urban site and in the suburban localities, and a bimodal pattern was noted in the forest site. The differences in the questing activity of this tick species may be associated with the environmental conditions prevailing in the urban heat islands. The diurnal course of vapour pressure characterised by two maxima and two minima, which is typical of non-urban areas, is not observed in the city. During the day, the vapour content in non-urban areas is significantly higher than in the city and decreases at night. Urban climate also differs in terms of precipitation, which is usually by several percent higher in urban areas than outside cities, with a shift of maximum precipitation from central districts towards peripheries [20].

In other parts of Poland, bimodal patterns of *I. ricinus* female activity are usually observed in forest localities, with maximum peaks in late spring (between May and mid-June) and in late summer (usually in September) [15]. In Europe, unimodal or bimodal patterns depending on microclimate conditions have been observed. From Slovakia, Pangrácová *et al.* [28] reported only unimodal activity of *I. ricinus* ticks (nymphs and adults) with a peak in May in north-eastern sites and in April in south-eastern sites. In contrast, bimodal patterns of tick activity were reported from various habitats in Hungary [29,30] and Slovenia [31].

Besides weather conditions, tick distribution and activity is influenced by the presence of hosts of the various tick developmental stages [32], which participate in the spread of *B. burgorferi* s.l. Maintenance of foci of infection with these spirochetes in tick populations is associated with occurrence in local ecosystems of animal species, which can be competent *B. burgdorferi* s.l. reservoirs, as active bacteraemia develops in their organisms [33-37].

The highest percentage of *I. ricinus* females infected with these bacteria was found in the city park in Chorzów (9.4%) and in the KO site at the city limits (5.9%), whereas the lowest percentage was noted in the forest KL site (3.12%) [9]. The predominant spirochete genospecies *B. burgdorferi* sensu stricto was identified in 5.0% of the examined ticks. Without showing distinct preferences, this spirochete genospecies infects two most important reservoir groups, i.e. small rodents and passerine birds [36-40].

The highest prevalence of spirochetes in *I. ricinus* females collected in the urban park may be related not only to the presence of competent reservoirs of these pathogens but also to the possibility of their rapid spread due to the persistent unimodal pattern of tick activity, which increases the chance of contact with the host. Transmission of *B. burgdorferi* from the vector into the host during feeding is the most effective and dominant mode of spread of this pathogen. Presumably, climatic conditions and large dust and gas pollution emissions within urban heat islands contribute to development and persistence of *Borrelia* spirochetes in ticks.

Alekseev and Dubinina [41] suggest that heavy metals present in contaminated environments and accumulated in tick organisms have an impact on metabolic processes, thereby leading to changes in the morphology and behaviour of these arthropods. In their investigations, abnormal *I. ricinus* females were more active and exhibited more severe infection with *B. burgdorferi* spirochetes than the normal specimens.

Occurrence of pathogen-infected ticks in recreational urban and suburban areas [42-47] poses a direct threat to human and animal health. Our study results confirming the presence of *I. ricinus* ticks and *B. burgdorferi* s.l. in urban and suburban habitats correspond with the incidence of Lyme borreliosis reported from these areas. The increase in the incidence of this zoonosis observed over the last 12 years implies existence of favourable conditions for spirochetes and their vectors in the area. In the neighbouring countries, the incidence varies from 11.3/100,000 inhabitants in 1999 to 19.2/100.000 in 2008 in Slovakia [48] and from 17.8/100,000 in 2000 to 37.3/100,000 in 2006 in Germany [49]. Similar trends towards an increased borreliosis incidence is observed in other European countries [50].

The seasonality of the prevalence of Lyme disease and other tick-borne diseases reported in our present and previous studies [2,15,51] as well as in the investigations conducted by other authors [48,49] is dependent on climatic and environmental conditions influencing the activity of pathogen vectors and reservoirs. An additional factor contributing to spread o Lyme disease is human behaviour, particularly recreational or occupational presence in tick habitats [51-53] and failure to comply with anti-tick prophylaxis principles [8,54].

The changes in the spread and abundance of *I. ricinus* [55] necessitate particular attention that should be paid to the effect of anthropogenic factors on the behaviour of these arthropods and the level of pathogen infection of ticks in urban and suburban recreational areas.

## Conclusions

Our investigations suggest that microclimate conditions and pollution within urban heat islands can affect the abundance and activity of *I. ricinus* nymphs and females, which most frequently attack humans and medium- and large-size animals. Low total abundance of both these tick stages exhibiting a unimodal pattern of female activity persisting for four months and a unimodal pattern of nymph activity was reported from the urban park, whereas the numbers of tick females with unimodal or bimodal activity and nymphs with unimodal activity increased in the localities situated on the outskirts and outside cities, respectively. The long-term activity of *I. ricinus* in the urban park indicates a high risk of host attacks, posed both to the residents and domestic and wild animals, and pathogen infections during the seasonal activity of this tick species. This type of tick activity can promote circulation of tick-borne pathogens in nature.
